# Regulation of somatostatin receptor 2 by proinflammatory, microbial and obesity-related signals in periodontal cells and tissues

**DOI:** 10.1186/s13005-018-0185-1

**Published:** 2019-01-04

**Authors:** Svenja Memmert, Anna Damanaki, Marjan Nokhbehsaim, Andressa V. B. Nogueira, Sigrun Eick, Joni A. Cirelli, Andreas Jäger, James Deschner

**Affiliations:** 1 Department of Orthodontics, Center of Dento-Maxillo-Facial Medicine, University of Bonn, Welschnonnenstr, 17 53111 Bonn, Germany; 2 Section of Experimental Dento-Maxillo-Facial Medicine, Center of Dento-Maxillo-Facial Medicine, University of Bonn, Bonn, Germany; 3 Department of Periodontology and Operative Dentistry, University Medical Center of the Johannes Gutenberg University, Mainz, Germany; 4 Department of Diagnosis and Surgery, School of Dentistry at Araraquara, Sao Paulo State University, UNESP, Araraquara, Brazil; 5 Department of Periodontology, Laboratory for Oral Microbiology, zmk bern, Zahnmedizinische Kliniken, Bern, Switzerland

**Keywords:** SSTR2, Inflammation, Fusobacterium nucleatum, Adipokines, Periodontitis

## Abstract

**Background:**

Periodontitis is a chronic disease characterized by a progressive and irreversible destruction of the tooth-supporting tissues, including gingiva and periodontal ligament (PDL). Microorganisms, such as *Fusobacterium nucleatum*, evoke an inflammatory host response, which leads to increased levels of inflammatory mediators, such as interleukin (IL)-1β. Periodontitis has been linked to obesity, and adipokines have been suggested to represent a pathomechanistic link. The hormone somatostatin (SST) exerts antiproliferative, antiangiogenetic, proapoptotic, anti-nociceptive and other effects through binding to its receptors, such as SSTR2. Therefore, the objective of the present study was to examine the regulation of SSTR2 in periodontal cells and tissues under inflammatory, microbial and obesity-related conditions.

**Methods:**

In-vitro, human PDL fibroblasts were exposed to IL-1β, *F. nucleatum*, leptin or visfatin. The SSTR2 regulation was assessed by real-time PCR and immunocytochemistry. In-vivo, the SSTR2 expression was analyzed in gingival biopsies of periodontally diseased and healthy subjects by real-time PCR and immunohistochemistry. Additionally, the SSTR2 expression was determined in gingival biopsies of rats with ligature-induced periodontitis, rats with diet-induced obesity, and periodontally and systemically healthy control animals. For statistical analyses, the Mann-Whitney-U test and ANOVA with post-hoc tests were applied (*p* < 0.05).

**Results:**

Exposure of PDL cells to IL-1β and *F. nucleatum* caused a significant SSTR2 upregulation by 2.6-fold and 6.4-fold, respectively. Additionally, leptin and visfatin increased significantly the SSTR2 gene expression by 3.0-fold and 2.8-fold, respectively. These stimulatory effects were also observed at protein level. SSTR2 expressions in human gingival biopsies from sites of periodontitis were significantly higher than those in healthy biopsies. Similarly, SSTR2 expression levels were significantly enhanced at periodontally-diseased sites in rat experimental periodontitis. Finally, the SSTR2 expression was significantly upregulated in gingival biopsies of obese rats as compared to normal weight control animals.

**Conclusions:**

Our study provides original insights into the SSTR2 regulation in cells and tissues of the periodontium. We demonstrate for the first time that proinflammatory, microbial and obesity-associated molecules result in an SSTR2 upregulation. Since SST has been shown to be antiproliferative, antiangiogenetic, and proapoptotic, our study suggests that SSTR2 might play a critical role in the aetiopathogenesis of periodontitis.

## Background

Periodontitis is a highly prevalent chronic inflammatory disease with social, psychological and physiological impacts. The disease is characterized by progressive and irreversible destruction of the tooth-supporting tissues, i.e. the periodontium, which comprises the gingiva, periodontal ligament (PDL), root cementum and alveolar bone [1, 2]. Microorganisms of the subgingival biofilm, such as *Fusobacterium nucleatum*, are essential for the initiation and progression of periodontitis [3–5]. The periodontopathogenic microorganisms, their constituents and products evoke an inflammatory host response, which leads to increased levels of inflammatory mediators, such as interleukin (IL)-1β, in gingival tissues and crevicular fluid (GCF). The inflammatory processes cause the release of matrix-degrading proteases and osteoclast-activating factors, which can ultimately result in attachment and tooth loss [3, 4, 6].

Periodontitis has been linked to a plethora of systemic diseases and conditions, such as type II diabetes, obesity and metabolic syndrome [7–10]. Although the exact underlying mechanisms for the interactions between periodontitis and these systemic diseases are only partially known, it has been suggested that adipokines might be a critical pathomechanistic link [11–13]. Obese patients are characterized by an increased amount of adipose tissue, which represents a complex endocrine and highly active metabolic organ. In the adipose tissue, adipocytes and other cells, such as leukocytes, secrete numerous bioactive molecules, which are collectively called adipokines [14–16]. Besides their function in the metabolic regulation, adipokines also regulate inflammatory and wound healing processes. Leptin and visfatin are adipokines with proinflammatory characteristics. Since the leptin secretion is proportional to the size and number of adipocytes, the plasma leptin level is increased in obese patients and decreased after weight loss [17]. As for leptin, the plasma level of visfatin, which is predominantly secreted by adipocytes and macrophages, is also enhanced in obese individuals [18, 19]. These adipokines can also be measured in the gingival tissues and GCF. Interestingly, their gingival levels seem to be altered in the presence of periodontitis, suggesting a local production and role of these adipokines in periodontal diseases [20–25].

The polypeptide hormone somatostatin (SST) antagonizes growth hormone (GH) and exerts antiproliferative, antiangiogenetic, proapoptotic, anti-nociceptive and other effects [26, 27]. Although very little is known about the presence and actions of SST in periodontal tissues, a few studies have detected SST-producing dendritic cells in the gingival epithelium and the subepithelial connective tissue [28–30]. SST can bind to five ubiquitously distributed G protein-coupled receptors (SSTR1–5), which mediate the aforementioned effects of SST [26]. Activation of SSTR2, which is one of the 5 receptors, results in a reduced expression of vascular endothelial growth factor (VEGF), insulin-like growth factor (IGF)-1 and their receptors, which all have been shown to be critical in periodontal health and repair [31–34]. Furthermore, SSTR2 could be a potential target for diagnostic strategies in periodontal bone infection and inflammation [35]. Deciphering the regulation of SSTR2 might be important for a better understanding of the aetiopathogenesis of periodontal diseases. Therefore, the objective of the present study was to examine the regulation of SSTR2 in periodontal cells and tissues under inflammatory, microbial and obesity-related conditions.

## Material und methods

### Culture and treatment of cells

PDL fibroblasts of caries-free and periodontal healthy teeth extracted for orthodontic reasons were harvested from 4 patients (mean age: 14.5 years, min-max: 11–19 years; gender: 3 male/1 female). Approval of the Ethics Committee of the University of Bonn (#117/15) as well as written and informed consent of the patients or legal guardians were given. Cells were cultured as previously described. Briefly, PDL tissue explants from the middle third of the roots were grown in Dulbecco’s minimal essential medium (DMEM, Invitrogen, Karlsruhe, Germany) supplemented with 10% fetal bovine serum (FBS, Invitrogen), 100 units penicillin and 100 μg/ml streptomycin (PenStrep Invitrogen) at 37 °C in a humidified atmosphere of 5% CO_2_ [36, 37]. For the experiments, PDL fibroblasts of passages between 3 and 5 were used at 80% confluence. One day prior to the experiments, the FBS concentration was reduced to 1%. Physiological concentrations of IL-1β (0.1–10 ng/ml; Calbiochem, San Diego, CA, USA), and *F. nucleatum* (ATCC 25586; optical density at wave length of 660 nm: 0.0125–0.05), leptin (1–10 ng/ml; R&D Systems, Minneapolis, MN, USA), and visfatin (30–300 ng/ml; Biomol, Hamburg, Germany) were applied as in our previous studies [21–23, 38–43]. *F. nucleatum* was inactivated via suspension in PBS (optical density at wave length of 660 nm = 1, equivalent to 1.2 × 10^9^ bacterial cells/ml) and ultrasonication of 160 W twice for 15 min. Inactivation of bacteria was checked by subcultivation on Schaedler agar plates in anaerobic conditions. PDL fibroblasts were exposed to the different stimulants for 1 d. Untreated cells served as control.

### Analysis of gene expression

The SSTR2 gene expression was analyzed via real-time polymerase chain reaction (RT-PCR) by using an iCycler iQ5 Detection System (Bio-Rad Laboratories, Munich, Germany). Briefly, RNA was extracted with a RNA extraction kit (RNeasy Protect Mini Kit, Qiagen, Hilden, Germany) and transcribed to cDNA with the iScript Select cDNA Synthesis Kit (Bio-Rad Laboratories). Afterwards, a 25 μl reaction mixture was prepared, containing 2.5 μl QuantiTect Primer Assay (Qiagen), 12.5 μl QuantiTect SYBR Green Master Mix (Qiagen) and 9 μl of nuclease free water as well as 1 μl of cDNA. The manufacturer’s protocol comprised a heating phase at 95 °C for 5 min for enzyme activation as well as 40 cycles of a denaturation step at 95 °C for 10 s and a combined annealing/extension step at 60 °C for 30 s. Glyceraldehyde-3-phosphate dehydrogenase (GAPDH) was used as an endogenous control.

### Analysis of intracellular protein levels

PDL fibroblasts were cultured on glass coverslips in the presence and absence of IL-1β, *F. nucleatum*, leptin or visfatin (Carl Roth, Karlsruhe, Germany) for 24 h. Afterwards, cells were fixed in 4% paraformaldehyde (Sigma-Aldrich, Munich, Germany) at pH 7.4 and room temperature for 10 min, washed with PBS (Sigma-Aldrich), and treated with 0.1% Triton X-100 (Sigma-Aldrich) for 5  min. Background staining was prevented by the application of serum block for 20 min (Dako, Hamburg Germany). After washing, cells were incubated with a rabbit polyclonal anti-SSTR2 antibody (10 μg/ml; R&D Systems) at 4 °C overnight. Goat anti-rabbit IgG-HRP (Dako) was applied as a secondary antibody for 45  min. Antibody binding was visualized by DAB chromogen (Thermo Fisher Scientific, Waltham, MA, USA) staining for 10  min at room temperature. Two washing steps with PBS (Sigma-Aldrich) were always performed between the incubation steps. Finally, cells were counterstained with Mayer’s Hematoxylin (Merck Eurolab, Dietikon, Switzerland) for 1  min and coverslipped with DePex mounting medium (Serva Electrophoresis, Heidelberg, Germany). Standardized photomicrographs were taken with an Axioskop 2 microscope (Carl Zeiss, Jena, Germany) equipped with an AxioCam MRc camera (Carl Zeiss) and the AxioVision 4.7 software (Carl Zeiss).

### Human biopsies

Gingival biopsies of 7 periodontally healthy donors (mean age: 22.1 years, min–max: 18–26 years; gender: 2 male/5 female) and 7 periodontally-diseased patients (mean age: 58.4 years, min–max: 29–81 years; gender: 5 male/2 female) were used for our experiments. These biopsies were obtained during wisdom tooth extraction and extractions for orthodontic or periodontal reasons in the Department of Oral Surgery of the University of Bonn [44]. Written and informed consent by the subjects and approval by the Ethics Committee of the University of Bonn (#043/11) were provided. Periodontal health or disease were assessed by gingival index (GI), probing pocket depth (PD), clinical attachment level (CAL), and radiographic bone loss. No clinical inflammation (GI = 0), a PD below 3 mm and neither clinical nor radiographic bone loss were categorized as periodontally healthy. Periodontitis patients were identified by sites with a GI > 1 and PD ≥ 5 mm and clinical as well as radiographic bone loss ≥3 mm, as previously described [21, 44, 45]. Smokers as well as patients taking any medication were excluded from the study.

The gingival biopsies were harvested as described above and then further analyzed by RT-PCR. In addition, the SSTR2 protein levels of the biopsies were studied by immunohistochemistry. Briefly, the gingival tissues were fixed with 4% paraformaldehyde (Sigma-Aldrich) for 2 d. Subsequently, the tissues were hydrated, then dehydrated in an ascending ethanol series (AppliChem, Darmstadt, Germany) and, subsequently, embedded in paraffin (McCormick Scientific, Richmond, IL, USA). Tissue slices of 3 μm thickness were cut and mounted on glass slides (Carl Roth). Finally, the slices were dried overnight, deparaffinized, rehydrated, and rinsed in TBS (TRIS und NaCl, MP Biomedicals, Illkirch, France; Merck KGaA, Darmstadt, Germany) for 10 min. Endogenous peroxidase was blocked with 0.3% methanol (AppliChem)/H_2_O_2_ (Merck Eurolab) solution for 10 min. Afterwards, an additional blocking step with serum block (Dako) for 20 min was carried out. Finally, the sections were incubated with a rabbit polyclonal anti-SSTR2 antibody (2.5 μg/ml; Research and Diagnostic Systems) in a humid atmosphere at 4 °C overnight. Goat anti-rabbit IgG-HRP (Dako) was used as a secondary antibody for 30  min, and the antibody binding was visualized by DAB chromogen (Thermo Fisher Scientific). Afterwards, the sections were counterstained with Mayer’s Hematoxylin (Merck Eurolab), and then imaged with an Axioskop 2 microscope (Carl Zeiss) equipped with an AxioCam MRc camera (Carl Zeiss) and the AxioVision 4.7 software (Carl Zeiss), as described above.

### Experimental periodontitis model

An experimental periodontits model was applied to analyze the expression of SSTR2 in gingival biopsies during the development of periodontal disease [46]. Thirty-two male adult Holtzman rats (average weight: 300 g) were assigned randomly to two experimental groups: control (sham-operated) and experimental periodontitis. Periodontitis was induced through cotton ligatures at the cervical area of the upper first molars. The ligatures were placed around the teeth, knotted mesially and left there for up to 20 d. Protocol followed the ARRIVE guidelines, and approval of the Ethical Committee on Animal Experimentation (protocol number: 23/2012) from the School of Dentistry at Araraquara, São Paulo State University – UNESP, was obtained. Animal housing was provided at the animal facilities of the School of Dentistry at Araraquara under controlled conditions (22–25 °C, 12 h light/dark cycle, standard laboratory diet and water ad libitum). Ten % ketamine hydrochloride (0.08 ml/100 g body weight) and 2% xylazine hydrochloride (0.04 ml/100 g body weight) were injected intramuscularly. Following 6 d, 8 d, 12 d, and 20 d, 4 animals per group were sacrificed by an anesthetic overdose. The maxillary jaws were harvested and, subsequently, the gingival tissues around the maxillary first molars were dissected for RNA extraction and RT-PCR (see above).

### Diet-induced obesity model

Ten male Wistar rats purchased from Charles River (Sulzfeld, Germany) were kept in a temperature (21 °C) and humidity (35%) controlled environment with a 12-h dark-light cycle. All animals received food and water ad libitum. All procedures were conducted according to the ethical standards of the University of Bonn (Az 87–51.04.2010.A394). All applicable international, national, and/or institutional guidelines for the care and use of animals were followed. The rats were equally divided into two groups. The animals of the test group were fed a normal diet until week 4 and, subsequently, a high-fat (25.1% fat), high sucrose (28.8% sucrose) diet (HFSD, sniff, Germany) until week 15. The animals of the control group were provided a normal diet for the entire 15 weeks. Animal weight was determined and documented weekly [47]. At week 15, the rats of the two groups were sacrificed for further analyses. The jaws were harvested and, subsequently, the gingival tissues were dissected for RNA extraction and RT-PCR (see above).

### Statistical analysis

Statistical analysis was carried out with the IBM SPSS Statistics software (Version 22, IBM SPSS, Chicago, IL, USA). Mean values and standard errors of the mean (SEM) were calculated for quantitative data. Experiments were done in triplicate and repeated at least twice. For statistical comparisons, the t-test, Mann-Whitney-U test or ANOVA followed by the post-hoc Dunnett’s test were used. Differences between groups were considered significant at *p* < 0.05.

## Results

### Regulation of SSTR2 gene expression by interleukin-1β, *F. nucleatum* and adipokines

**Fig. 1:**
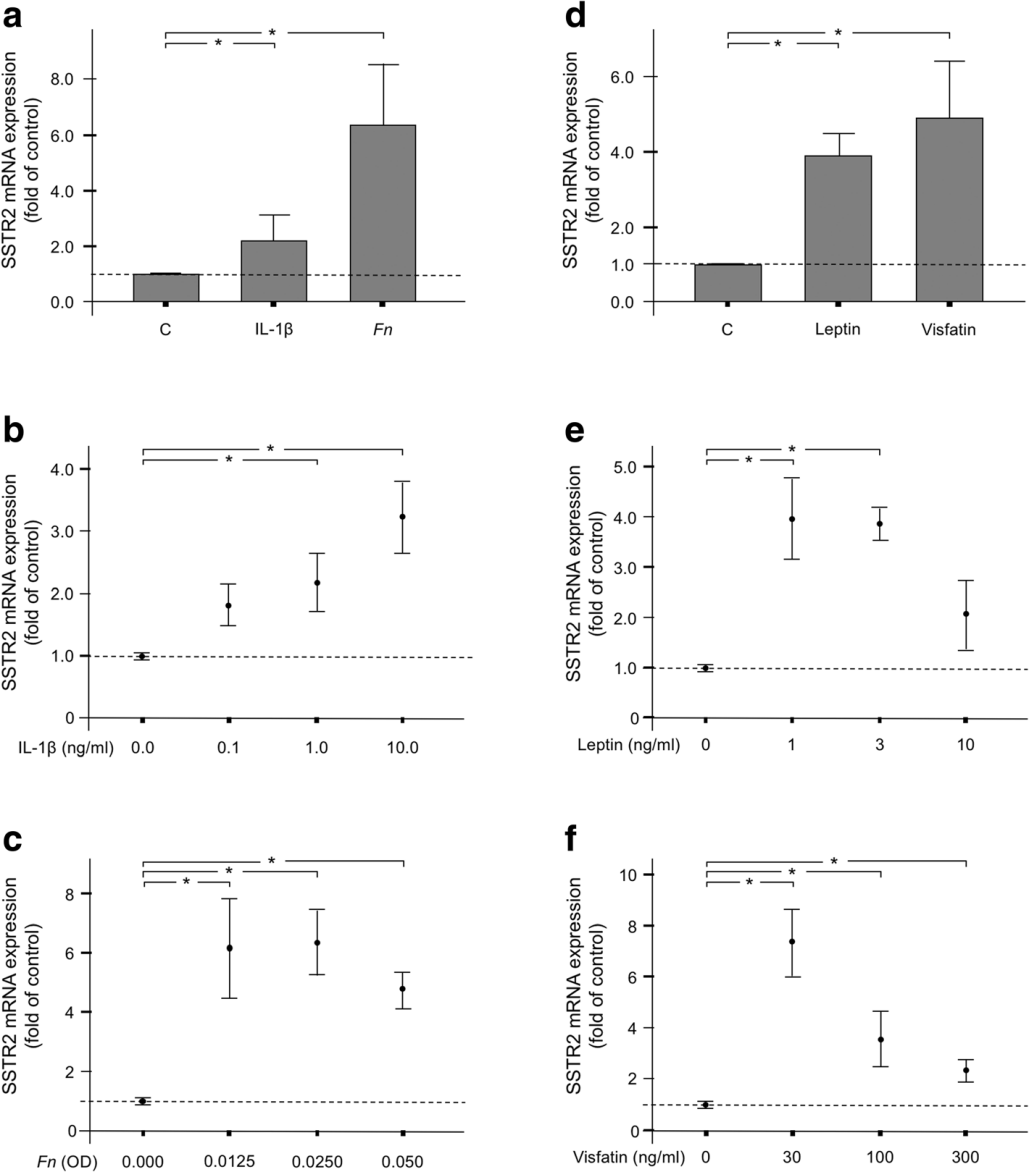
(**a**) SSTR2 expression in the presence or absence of IL-1β (1 ng/ml) or *F. nucleatum* (OD: 0.025) at 1 d. Untreated cells served as control. Mean ± SEM (*n* = 6), * significantly (*p* < 0.05) different from control. (**b**) and (**c**) Effects of various concentrations of IL-1β (0.1–10 ng/ml) or *F. nucleatum* (OD: 0.0125–0.05) on the SSTR2 expression at 1 d. Untreated cells served as control. Mean ± SEM (*n* = 6); * significantly (*p* < 0.05) different from control. (**d**) SSTR2 expression in the presence or absence of leptin (3 ng/ml) or visfatin (100 ng/ml) at 1 d. Untreated cells served as control. Mean ± SEM (*n* = 6), * significantly (*p* < 0.05) different from control. (**e**) and (**f**) Effects of various concentrations of leptin (1–10 ng/ml) or visfatin (30–300 ng/ml) on the SSTR2 expression at 1 d. Untreated cells served as control. Mean ± SEM (*n* = 6); * significantly (*p* < 0.05) different from control

First, we sought to analyze whether proinflammatory, microbial and obesity-related signals have an impact on the expression of SSTR2 in PDL fibroblasts. Exposure of cells to IL-1β caused a significant SSTR2 upregulation by 2.6-fold, as shown in Fig. 1a. Further analysis revealed that the stimulatory effect of IL-1β was dose-dependent, with the highest dose resulting in the most pronounced SSTR2 expression (Fig. 1b). In addition, the periodontitis-associated microorganisms *F. nucleatum* induced a significant increase in SSTR2 expression by 6.4-fold and this stimulatory action of *F. nucleatum* was observed over a wide range of concentrations (Figs. 1a and c). Next we sought to examine whether the SSTR2 expression is also regulated by the proinflammatory adipokines leptin and visfatin. Interestingly, leptin and visfatin induced a significant SSTR2 upregulation by 3.9-fold and 4.9-fold, respectively, as shown in Fig. 1d. Further experiments demonstrated that the stimulatory effects of the two adipokines decreased with increasing concentrations (Figs. 1e and f).

### Regulation of SSTR2 protein levels by interleukin-1β, *F. nucleatum* and adipokines

**Fig. 2:**
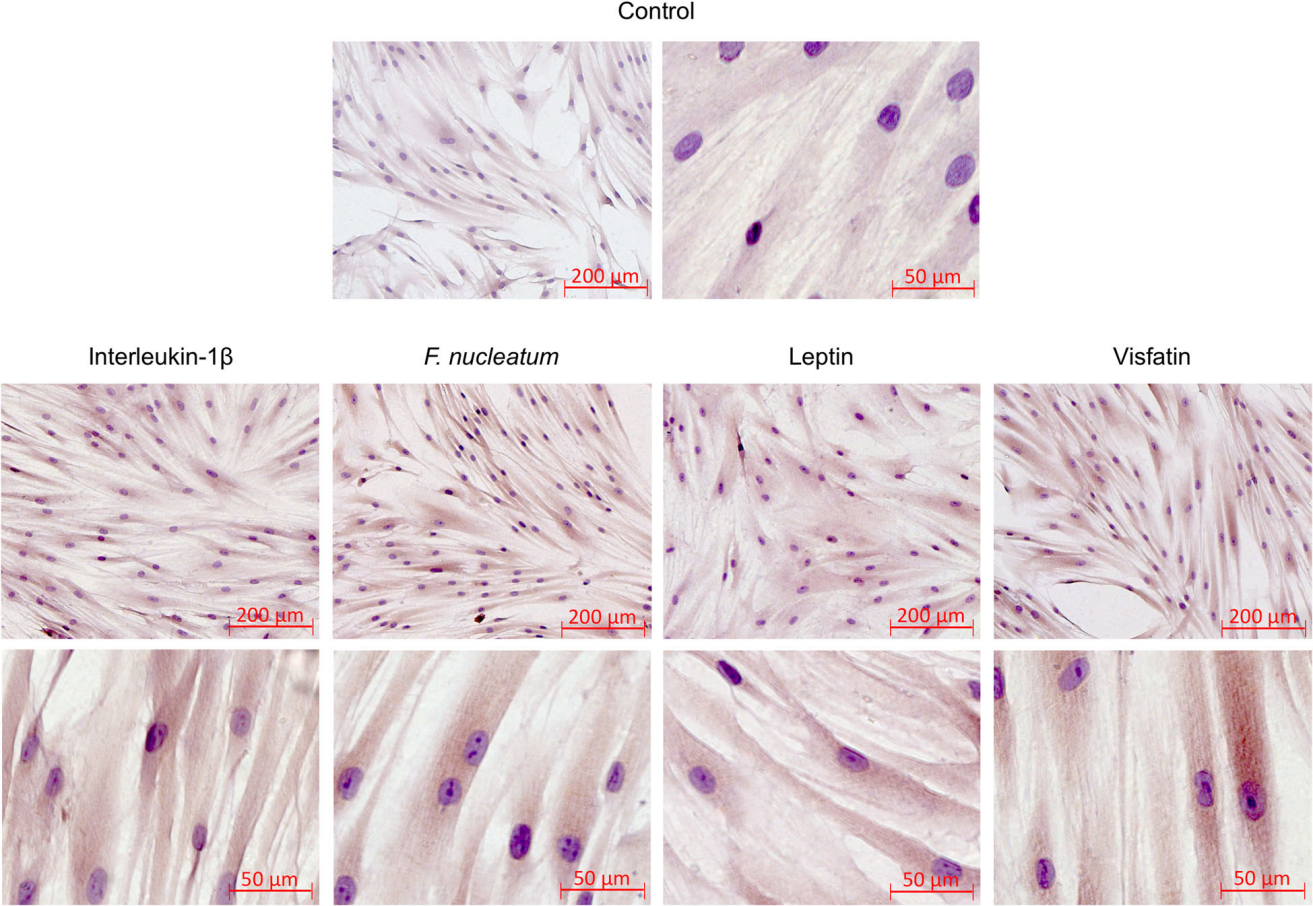
Effects of IL-1β (1 ng/ml), *F. nucleatum* (OD: 0.025), leptin (3 ng/ml) or visfatin (100 ng/ml) on SSTR2 protein levels in PDL fibroblasts at 1 d, as analyzed by immunocytochemistry. Untreated cells served as control. Experiments were performed in triplicates and representative images of cells from one donor are shown

We then analyzed by immunocytochemistry whether the stimulatory actions of the proinflammatory, microbial and obesity-related signals can also be observed at protein level. As depicted in Fig. 2, exposure of PDL fibroblast with IL-1β*, F. nucleatum*, leptin or visfatin resulted in an enhanced immunoreactivity against SSTR2, as compared with control cells. SSTR2 protein was found to be equally distributed throughout the cell cytoplasm (Fig. 2).

### Regulation of SSTR2 in human and rat gingival biopsies

**Fig. 3:**
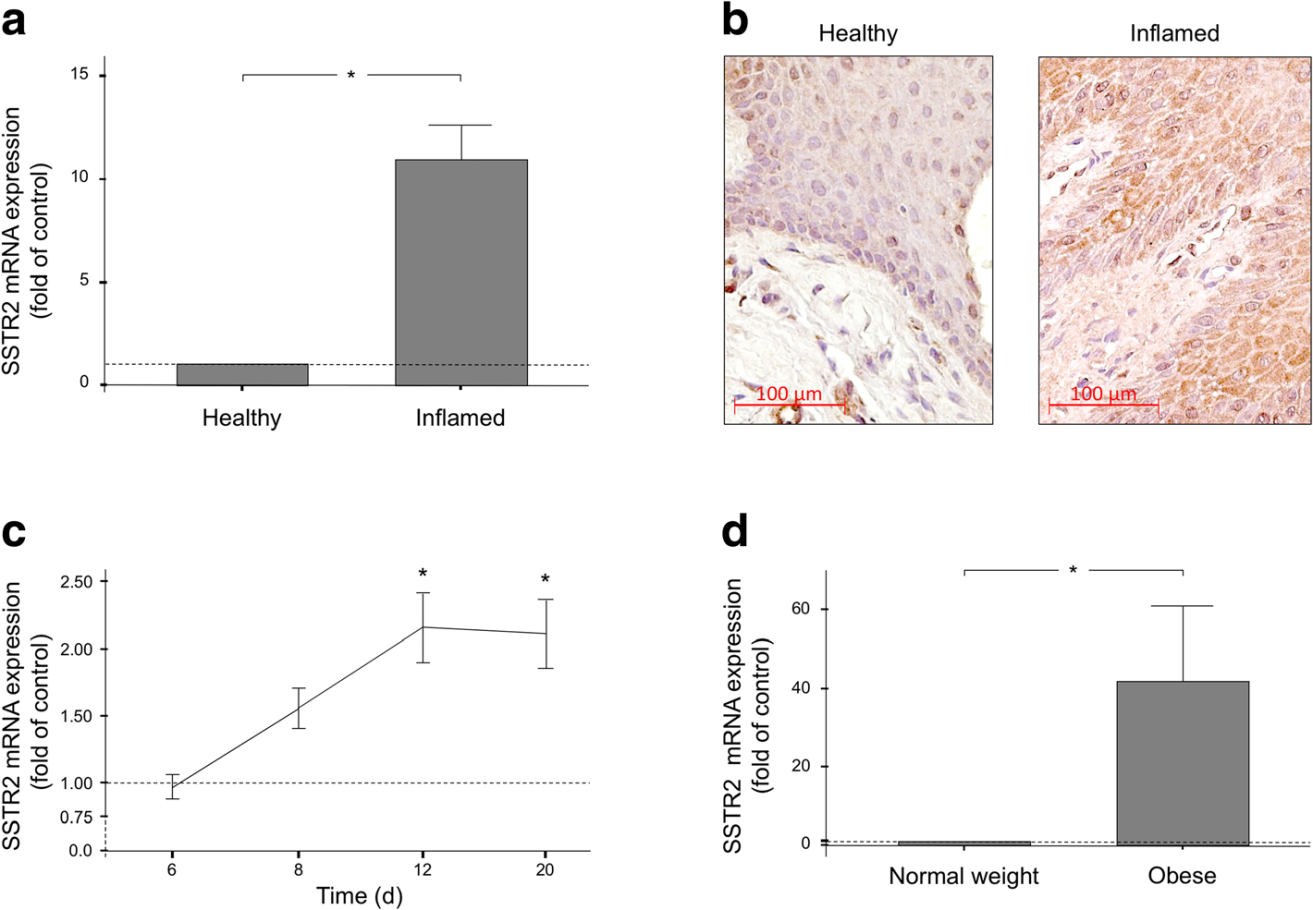
(**a**) SSTR2 expression in human gingival biopsies from periodontally inflamed and healthy sites. Mean ± SEM (*n* = 7 per group); * significant (*p* < 0.05) difference between groups. (**b**) SSTR2 protein in human gingival biopsies from periodontally inflamed and healthy sites, as analyzed by immunohistochemistry. Representative images of biopsies of one donor per group are shown. (**c**) SSTR2 expression in gingival biopsies of rats with ligature-induce periodontitis at 6 d, 8 d, 12 d and 20 d, as compared to control animals. Mean ± SEM (*n* = 4/group and time); * significantly (*p* < 0.05) different from control animals. (d) SSTR2 expression in gingival biopsies of obese and normal-weight rats. Mean ± SEM (*n* = 5); * significant (*p* < 0.05) difference between groups

Finally, we sought to investigate whether the SSTR2 regulations, as observed in our in-vitro experiments, could also be found in a more complex environment. Therefore, human biopsies from periodontally-healthy and periodontitis sites were obtained and, subsequently, analyzed for potential differences in the SSTR2 expression and protein levels by RT-PCR and immunohistochemistry. As expected, the SSTR2 expression in gingival biopsies from sites of periodontitis was significantly higher than that in healthy biopsies (Fig. 3a). In parallel, a stronger immunoreaction to SSTR2 was found in inflamed biopsies as compared to the control tissues, but control tissues also showed weak but continuous immunoreaction to SSTR2 as expected (Fig. 3b). Similarly, SSTR2 expression levels were also significantly enhanced at periodontally-diseased sites in a rat model of experimental periodontitis. The time-course analysis revealed that the SSTR2 expression increased in the presence of plaque accumulation until day 12 and remained at this significantly enhanced level until the end of the in-vivo study (Fig. 3c). Lastly, we also studied the effects of obesity on the SSTR2 expression in gingival biopsies in a rat diet-induced obesity model. As analyzed by RT-PCR, the SSTR2 expression was significantly upregulated in gingival biopsies of obese rats as compared to normal-weight control animals (Fig. 3d).

## Discussion

The present study provides novel insights into the regulation of SSTR2 in periodontal cells and tissues. Our in-vitro and in-vivo experiments show that proinflammatory, microbial and obesity-associated molecules cause a strong upregulation of SSTR2. Since SST has antiproliferative, antiangiogenetic, and proapoptotic effects, our findings suggest that SSTR2 might play a critical role in periodontal diseases.

There are only few studies, which have reported on SST and its receptors in periodontal tissues [28–30]. However, the role of the SST/SSTR system in periodontal health and disease has yet to be elucidated. We therefore exposed periodontal fibroblasts to IL-1β, which has been shown to be increased at periodontally inflamed sites, and to the periodontopathogen *F. nucleatum*, which is a gram-negative anaerobic microorganism associated with gingivitis and periodontitis [5, 6]. Our experiments revealed that the SSTR2 expression was enhanced under these inflammatory and microbial conditions, indicating that the SST/SSTR system might play an important role in periodontal inflammation and infection. Interestingly, a study on human corneal epithelial cells and an immortalized epithelial cell line from human conjunctiva also demonstrated increased SSTR2 expressions in response to proinflammatory cytokines, such as IL-1ß, and bacterial components, such as lipopolysaccharides or peptidoglycans [48]. These findings concur well our data and emphasize the potential role of the SST/SSTR2 system in human physiology and pathophysiology. Moreover, since periodontitis is linked with obesity, we also incubated periodontal fibroblasts with the proinflammatory adipokines leptin and visfatin, whose plasma levels are increased in obese individuals [17, 19]. These adipokines are also produced locally in periodontal tissues and altered at periodontally inflamed sites, as studies by our group and other investigators have shown [11, 21–23, 49]. Like IL-1β and *F. nucleatum*, these adipokines also induced an upregulation of SSTR2, underlining their proinflammatory characteristics and indicating a potential mechanism whereby adipokines could contribute to periodontal destruction. To our best knowledge, our study is the first one which links visfatin with SSTR2. The stimulatory effects of IL-1β, *F. nucleatum*, leptin and visfatin on SSTR2 were also observed at protein level, indicating that our transcriptional findings are physiologically relevant. In our experiments, the concentrations of the stimulants were in the physiological range and consistent with the doses of previous studies [44, 45, 49–51].

In order to validate our in-vitro findings in a more complex environment, we also studied the SSTR2 expression in human biopsies from periodontally healthy subjects and from periodontitis patients. As demonstrated by RT-PCR and immunohistochemistry, SSTR2 levels were strongly enhanced at sites of periodontitis as compared to control sites, thereby confirming the findings of our in-vitro study, where IL-1β was used to mimic periodontal inflammation and *F. nucleatum* was applied to simulate periodontal infection. In a rat experimental periodontitis model, we also investigated the time course of SSTR2 expression in gingival biopsies. In this model, ligatures were used to induce periodontitis, which resulted in significant alveolar bone resorption, as presented in our previous study [46]. The in-vivo study supported our in-vitro data and the findings from the human biopsies by demonstrating that microbial plaque accumulation finally results in an upregulation of SSTR2. Interestingly, a recent study focused on the expression of SSTRs in the jejunums of *Cryptosporidium parvum*-infected rats [52]. The investigators found a significant increase in the SSTR2 expression in the inflamed jejunum following infection, indicating that SSTRs may regulate inflammatory pathways in rat intestine. These findings are in accordance with our results showing increased SSTR2 levels at inflamed sites. By contrast, SSTR2 was found to be downregulated in a rat lipopolysaccharide-induced periodontitis model [53]. However, in this model, periodontitis was caused by injection of *Escherichia coli* lipopolysaccharides into the palatal gingival sulci. In our model, periodontitis was established through cotton ligatures at the cervical area of the upper first molars, which usually results in the accumulation of a complex multispecies biofilm. We think that this model better simulates the plaque-induced development of periodontitis, as observed in humans, and the differences in the applied models might explain the opposite results. Moreover, our in-vivo findings are in line with our in-vitro data, thereby supporting our in-vivo observation. Taken together, our in-vitro and in-vivo results suggest a potential role of SSTR2 in the aetiopathogenesis of periodontitis.

Furthermore, we studied possible effects of obesity on the SSTR2 expression in gingival biopsies in a rat diet-induced obesity model [47]. This in-vivo study revealed that obesity leads to an enhanced SSTR2 expression in gingival tissues, thereby supporting our in-vitro experiments with adipokines. Interestingly, acute central leptin infusion on the SSTR-effector system in the brain of rats also resulted in increased SSTR2 gene and protein levels [54], indicating a regulatory role of leptin on this receptor.

Although the present study suggests a potential role of SSTR2 in the aetiopathogenesis of periodontal disease, we have not examined the actions of SST itself and the regulation of other SSTRs. Therefore, future studies should also focus on the actions of SST and on the roles of other SSTRs in periodontal cells and tissues. Additionally, further studies should analyze the role of the SST/SSTR system in different periodontal cells like gingival fibroblasts.

In the present study, cells were exposed to the periodontopathogen *F. nucleatum*. However, periodontitis is a chronic inflammatory disease caused by a highly complex biofilm [5]. Therefore, further studies should also examine the SSTR2 regulation by other microorganisms and their combinations. In addition, like the microbial biofilm, the inflammatory processes are also complex and involve a plethora of pro- and antiinflammatory mediators [6]. Whether these molecules exert the same or opposite effects, as compared to IL-1β, has yet to be determined.

It has been suggested that adipokines, such as leptin and visfatin, could represent a pathomechanistic link in the interactions between periodontitis and obesity [11]. It is possible that adipokines contribute to the aetiopathogenesis of periodontitis, as a lot of studies suggest [11–13]. However, adipokines are also produced locally by periodontal cells and tissues [21–23]. Interestingly, the local production of these adipokines is regulated by inflammatory and microbial signals, suggesting that adipokines also play a role in periodontal inflammation and destruction in the absence of obesity. Future studies should also examine the actions of other adipokines, such as resistin or apelin, on the SST/SSTR2 system in the periodontium. Moreover, further studies should be dedicated to the intracellular signaling pathways, which are involved in the observed SSTR2 upregulation.

## Conclusions

Our study provides original insights into the SSTR2 regulation in cells and tissues of the periodontium. We demonstrate for the first time that proinflammatory, microbial and obesity-associated molecules result in an SSTR2 upregulation. Since SST has been shown to be antiproliferative, antiangiogenetic, and proapoptotic, our study suggests that SSTR2 might play a critical role in the aetiopathogenesis of periodontitis.

## Abbreviations

CAL: Clinical attachment level
DMEM: Dulbecco’s minimal essential medium
FBS: Fetal bovine serum
GAPDH: Glyceraldehyde-3-phosphate dehydrogenase
GCF: Gingival crevicular fluid
GH: Growth hormone
GI: Gingival index
IGF: Insulin-like growth factor
IL: Interleukin
PD: Probing pocket depth
PDL: Periodontal ligament
RT-PCR: Real-time polymerase chain reaction
SST: Somatostatin
SSTR: Somatostatin receptor
VEGF: Vascular endothelial growth factor
